# “Bacterial Toxins” Section in the Journal *Toxins*: A Fantastic Multidisciplinary Interplay between Bacterial Pathogenicity Mechanisms, Physiological Processes, Genomic Evolution, and Subsequent Development of Identification Methods, Efficient Treatment, and Prevention of Toxigenic Bacteria

**DOI:** 10.3390/toxins10010044

**Published:** 2018-01-18

**Authors:** Michel R. Popoff

**Affiliations:** Institut Pasteur, Unité des Bactéries Anaérobies et Toxines, 25 Avenue du Docteur Roux, 75015 Paris, France; michel-robert.popoff@pasteur.fr; Tel.: +33-1-4568-8307; Fax: +33-1-4061-3123

Toxins are powerful pathogenicity factors produced by certain bacteria, fungi, animals, and plants which mediate drastic interactions of these pathogens on the organism host. Notably, bacterial toxins were the first compounds which were identified as responsible for severe bacterial diseases in man and animals. Numerous studies and publications were dedicated to bacterial toxin characterization and deciphering of their mechanisms of action. The “Bacterial Toxins” section of the journal Toxins is entirely devoted to this topic. Classically, bacterial toxins are divided into exotoxins and endotoxins. While endotoxins are membrane compounds of Gram-negative bacteria which elicit an inflammatory response in host, exotoxins are secreted proteins which act locally and at distance of the bacterial colonization site. Specific factors produced by invasive bacteria, and called virulence factors, are related to exotoxins and some of them share similar enzymatic activities to those of exotoxins. However, in contrast to exotoxins, the virulence factors are directly injected into the cell where the bacterium is attached by specific secretion types, such as the type III secretion system, and their activity is restricted to the attacked cell. Numerous works, publications, and meetings have been related to these fascinating toxin proteins which display multiple activities and functions; such an example is the Sourcebook of Bacterial Protein Toxins [1] or the diverse special issues of the journal Toxins. Indeed, bacterial toxins show an extreme diversity regarding their size (from 15 to more than 2700 amino acids), mode of secretion (different bacterial secretion types), structure (mono, binary, ternary, or multiple complex proteins), membrane/intracellular receptor recognition, enzymatic activity, or specific mode of action such as pore-forming activity.

In the last decades, a great effort has been done to unravel the mechanisms of action of these very active proteins able to induce so severe symptoms in higher organisms. *Clostridium perfringens* alpha toxin was the first bacterial toxin identified as developing an enzymatic activity consisting of phospholipase C activity at the cell membrane surface [2]. Then, diphtheria toxin (DT) was the first toxin that was characterized to exert a novel enzymatic activity intracellularly [3,4]. Thereby, DT modifies the elongation factor-2 (EF-2) by ADP-ribosylation, leading to inhibition of cell protein synthesis and cell death. ADP-ribosylation was then recognized to be shared by numerous other intracellularly-active toxins. Other bacterial toxins develop an enzymatic activity commonly used by many cellular or bacterial enzymes, such as protease, glucosylase, DNase, or RNase, but towards a specific intracellular target promoting specific effects. This is the case of clostridial neurotoxins which specifically cleaves SNARE proteins in target neuronal cells, thus inhibiting the evoked release of neurotransmitters. Since bacterial toxins interact with specific cellular compounds and alter specific cellular processes, they are highly relevant tools not only to better understand pathogenicity mechanisms, but also to unravel physiological processes. An emblematic example is that of *Clostridium botulinum* C3 exoenzyme, which was characterized as an ADP-ribosyltransferase of a novel G-protein of unknown function, termed Rho, from the Ras superfamily. C3 exoenzyme was found to break the actin filaments in cultured epithelial cells and this opened the door to understanding the regulation of actin filament polymerization via the small GTPases of the Rho family [5]. Therefore, bacterial toxins are at the frontier of various disciplines including bacteriology, cellular biology, molecular biology, structural biology, biochemistry, genetics, immunology, and vaccinology. The diverse properties of bacterial toxins are well documented, notably in the multidisciplinary special issues of the journal *Toxins*.

More recently, novel advances have been performed in bacterial toxins which have been mainly facilitated by the development/improvement of molecular technologies. Indeed, whole genome sequencing of saprophytic and pathogenic bacteria with subsequent genomics analysis allowed the identification of novel toxins and the exploration of their spreading and evolution in the bacterial world. For example, typhoid toxin was initially suspected in cells invaded by *Salmonella typhi* and was subsequently characterized by genomic analysis and crystal structure, which revealed a novel toxin organization consisting of two distinct enzymatic subunits and five binding subunits. Typhoid toxin seems to have evolved from assembly of several ancestor toxin genes spread in other bacteria, such as cytolethal distending toxins and pertussis toxin [6]. The rapidly increasing progress in the whole genome sequencing of microorganisms would probably result in identification of novel toxins and in-depth understanding of their evolution. Moreover, refinement of the crystal structure investigations allowed unravelling the 3D structure of large or complex toxins, such as the whole structure of the large clostridial glucosylating toxin A from *Clostridium difficile* [7] or botulinum progenitor toxin complex [8]. However, many aspects of these multifunctional toxin proteins remain to be discovered. Albeit cellular receptors have been identified for some toxins, they are largely unknown for many others. Strategic features, such as toxin dissemination into the host to target cells, interaction with the cell membrane, crossing the phospholipid bilayer, intracellular trafficking, and precise mechanisms of cell alteration are still under investigation for many toxins.

In addition to their interest in fundamental science, bacterial toxins are key players in various applied developments, including tools for diagnosis, prevention, and therapy of diseases, due to toxigenic bacteria. Indeed, the diagnosis of several diseases is based on the detection and identification of toxins in biological and/or environmental samples, such as foods. The development of sensitive and rapid in vitro methods of toxin characterization are still in progress using new or improved technologies, such as mass spectrometry, refined ELISA, or fluorescent techniques. Since the pioneering historical works, detoxified toxins (anatoxins) were shown to elicit a solid preventive response against diseases due to toxigenic bacteria. Anatoxins are among the most efficient vaccines against bacterial diseases. Recombinant toxin subunits, which are biologically inactive, but retain the immunogenicity, offer the advantage to be safer than the classical detoxified toxins. Development of efficient toxin inhibitors are of major importance (see the special issue of *Toxins* edited by H. Barth). Although bacterial toxins are very poisonous compounds, some of their properties have powerful therapeutic applications. The most representative example is that of botulinum toxins which are used for their anti-cholinergic effects. Thereby, the most potent toxins are the drugs which have the most numerous medical indications from the treatment of dystonia, strabismus, hypersecretory activity of cholinergic glands, urinary bladder dysfunction, pain, cosmetology, etc. Botulinum toxins are mainly used as wild-type purified proteins, but engineering of these molecules is in progress to exploit specific properties of these toxins, such as therapeutic effects on sensitive neurons and the treatment of pain [9]. Recombinant toxins have also been engineered to specifically kill malignant cells (immunotoxins), or to transport therapeutic compounds into specific host compartments hardly accessible by routine administration pathways, such as the central nervous system. In addition, toxin development concerns not only medical applications, but also broad technical innovations. Thereby, the specific pore-forming activity of *Staphylococcus aureus* alpha toxin is used in novel processes of DNA sequencing [10].

Bacterial toxins are an increasing field of interest for scientists, health professionals, teachers, and students from various disciplines. The journal *Toxins*, which gathers toxin articles in specific sections and special issues, is quite appropriate to receive high-quality manuscripts, reviews, editorials, comments, and to promote fruitful discussions in the scientific community. We hope that *Toxins* will be more and more attractive to receive original and pertinent submissions.
